# Sociodemographic Factors and Neighborhood/Environmental Conditions Associated with Social Isolation Among Black Older Adults

**DOI:** 10.1177/08982643221118427

**Published:** 2022-09-23

**Authors:** Harry O. Taylor, Kazumi Tsuchiya, Ann W. Nguyen, Collin Mueller

**Affiliations:** 1 7938 University of Toronto, Toronto, ON, Canada; 2 7938 University of Toronto, Toronto, ON, Canada; 3 2546 Case Western Reserve University, Cleveland, OH, USA; 4 1068 University of Maryland, College Park, MD, USA

**Keywords:** African Americans, environment, neighborhoods, social support

## Abstract

**Objectives:** To investigate sociodemographic factors and neighborhood/environmental conditions associated with social isolation (SI) among Black older adults. **Methods:** We utilized data from the 2014 and 2016 Leave-Behind Questionnaire from the Health and Retirement Study (HRS LBQ) among those who self-identified as Black (*N* = 2.323). Outcome variables for our study included SI from adult children, other family members, friends, disengagement from social participation and religious services, being unmarried, and living alone. These indicators were also combined into an overall SI index. Critical predictors included gender, age, household income, education, employment status, neighborhood cohesion, neighborhood disorder, urbanicity, and region of residence. **Results:** Sociodemographic factors of gender, education and household income were significantly associated with SI indicators. Additionally, some neighborhood/environmental conditions were associated with SI indicators. **Discussion:** SI was found to be patterned by sociodemographic factors. These results can be used to develop effective interventions to mitigate SI among Black older adults.

## Introduction

Social isolation (SI) is a complex multidimensional construct. SI is defined as an objective condition in which an individual has limited to non-existent social contact among family members, friends, and also is disengaged from social participation and religious services (Taylor, 2020). SI has been previously operationalized as an index (Berkman & Syme, 1979; Cudjoe et al., 2020) and often measures the frequency of socializing with family members and friends, frequency of social participation, frequency of religious participation, living arrangements, and marital status (Taylor, 2020; Taylor et al., 2019). Approximately 1 out of 4 older adults is considered socially isolated (Chatters et al., 2018; Cudjoe et al., 2020), with prevalence estimates of isolation ranging between 15 and 40% of older adults in the US (Elder & Retrum, 2012).

SI is a chronically stressful condition associated with worse physical health and chronic conditions including cancer, hypertension, diabetes, and cardiovascular disease (National Academies of Sciences, 2020; Taylor, 2021; Tomaka et al., 2006). SI is also associated with worse mental health and greater cognitive decline and impairment (National Academies of Sciences, 2020; Nguyen et al., 2020; Taylor et al., 2018). Furthermore, recent meta-analysis studies have found those who are socially isolated have a 29% increased likelihood of mortality compared to those who are not, and that the mortality effects of SI are equivalent to smoking 15 cigarettes per day (Holt-Lunstad et al., 2010, 2015).

Previous studies have found numerous factors related to SI among older adults including demographic factors (e.g., low income or low education, belonging to a cultural or racial minority group, male gender) and neighborhood and environmental contextual factors including living in neighborhoods which lack meaningful activities, are unsafe, or have inaccessible built environment for older adults (Cudjoe et al., 2020; Elder & Retrum, 2012; Nicholson, 2012). This is important to consider given neighborhood/environmental conditions are the systems in which we are born, live, work, age, and die (World Health Organization, 2010). Additionally, these neighborhood/environmental contexts shape our relationships and interactions with family members and friends as well as how we engage with institutions and participate in social/group activities. By not accounting for neighborhood/environmental conditions, we are missing crucial elements that could have a significant influence on SI for all communities and populations. Nevertheless, to the investigators’ knowledge, there are only a handful of studies that have examined sociodemographic and neighborhood/environmental conditions associated with SI among Black older adults (Adams et al., 1989; Taylor et al., 2016, 2019).

### Importance of Studying Social Isolation Among Black Older Adults

Studying sociodemographic and environmental factors associated with SI among Black older adults specifically is important for numerous reasons. First, the population of Black older adults living in the United States is growing at a fast rate. From 2019 to 2040, it is estimated there will be an 80% increase in the population of Black older adults in the United States (from 4,979,133 in 2019 to 8,970,575 by 2040; Administration on Aging, 2021a, 2021b). While this statistic is positive and illustrates more Black older adults are living to older ages, many members of this population may be aging alone. In the coming years, it is projected that 12.6% of older Black men (or approximately 2.7 million) and 15.1% of older Black women (or approximately 3.3 million) will be kinless (not having a spouse or partner, nor any children) by 2060 (Verdery & Margolis, 2017). This would place a substantial proportion of Black older adults at greater risk for experiencing SI.

Second, given the cascading impacts of structural racism across the life course, many Black older adults navigate multiple challenging life circumstances that may place them at greater risk for SI via interpersonal, institutional and neighborhood factors, or by-products of racism (Bailey et al., 2017; LaFave et al., 2022). Black Americans are more likely to live in neighborhoods with concentrated disadvantage (e.g., poverty, unemployment) due to racial segregation driven by structural racism (Williams & Collins, 2001). Hence, Black older adults are more likely to live in poverty, obtain lower educational attainment, and have a greater likelihood to be either divorced, separated, or never married, and have worse physical health outcomes in comparison to the general population of older adults (Administration on Aging, n. d.; Chatters et al., 2021; LaFave et al., 2022; Williams & Collins, 2001). These factors are related to SI among the general population of older adults; however, there is extremely limited research which has examined if and/or how these factors are related to SI among Black older adults.

It is imperative to conduct within-group analyses of sociodemographic factors and neighborhood/environmental conditions for SI solely among Black older adults. Most studies examining factors associated with SI use race as a covariable in statistical models; however, this type of analysis only demonstrates if there is a significant racial difference in SI. If there are statistically significant racial differences in SI, this type of analysis does not tell us what is associated with the difference, or where or why these differences exist in the first place (Taylor, 1985). Alternatively, if there are no significant racial differences in SI among older adults, we may prematurely assume that there is no racial/ethnic variation in the risk factors for SI among older adults.

Furthermore, only using race as a covariable is not useful for examining the heterogeneity of lived experiences among Black older adults and may obscure potential significant within-group differences. Black older adults are not monolithic (Jackson et al., 2004; Taylor, 1985), and it is important to understand which factors are associated with SI within this population. *Said another way, there is a robust literature which illustrates potential risk factors that contribute to SI among older adults, however, additional research is needed to determine which of these factors are particularly salient for Black populations*. This information is critical in the development of evidence-based interventions which seek to ameliorate SI among Black older adults.

### Previous Research on Social Isolation Among Black Older Adults

To date, the investigators are aware of only three empirical studies which examine the prevalence and correlates of SI among Black populations (Adams et al., 1989; Taylor et al., 2016, 2019). In one of the first papers in this area, Adams et al. (1989) operationalized SI by examining kin and non-kin interactions. Factors that were associated with more SI from kin were having higher education, shorter length of residence in the respondents’ homes, fewer close relatives, less community participation, and worse functional status. Greater SI from non-kin was correlated with having lower media use and community participation, worse functional status and perceived health, and reporting fewer chronic health problems.

More recent studies of SI among African American families and African American older adults used the National Survey of American Life (NSAL), a nationally representative survey of African Americans, Black Caribbeans, and non-Hispanic Whites (Jackson et al., 2004; Taylor et al., 2016, 2019). Taylor et al. (2019) examined individual indicators of SI among Black older adults which included not having any contact with neighbors, friends, or family members, and not participating in neighborhood or religious groups, being single or not involved in a romantic relationship, and having no children. They found that women were more likely to be unmarried but less likely to be isolated from their religious congregational members and family members. Both education and income were associated with isolation, where those with more education were more likely to be isolated from their neighborhood groups and more likely to live alone, and those with higher income are less likely to live alone and less likely to be unmarried and not involved in a romantic relationship. These findings help illustrate the dynamic nature of SI among Black older adults, as different facets of SI are subsequently associated with different sociodemographic factors.

### Neighborhood Environments, Social Isolation, and Black Americans

Understanding how neighborhood environments influence SI specifically among Black older adults is very important given the United States is a highly segregated country by race and ethnicity. Black populations, including Black older adults, frequently reside in racially segregated communities that have greater built environmental hazards and physical degradation. Additionally, Black communities often have limited social, political, and economic resources in comparison to non-Hispanic White communities (Chatters et al., 2021; Redwood et al., 2010; Ross & Mirowsky, 2001; Williams & Collins, 2001). These environments that Black Americans often reside in are a result of a history of residential segregation through redlining, under-funding, and the gentrification of Black communities (Bailey et al., 2017; Chatters et al., 2021; Crewe, 2017).

Black older adults who are living in marginalized communities (e.g., perceived social and physical neighborhood conditions) may experience greater SI. For example, they may be afraid and unwilling to attend community services or to meet with friends and family that live in the community because of fear of crime, a lack of trust in the community, or due to limited transportation services that Black older adults can access (Klinenberg, 2001, 2005). On the other hand, it is also possible that Black older adults living in these areas may experience less SI. This is because there could be heightened efforts among residents to come together and collectively address the issues in their community. For example, a study by Schieman (2005) found that greater neighborhood disadvantage was positively associated with greater support given and received among Black women. However, there has not been research documenting the impacts of neighborhood conditions on the various dimensions of SI among Black older adults.

### Urbanicity, Region of Residence, Social Isolation, and Black Older Adults

Overall, there has been very little research examining urbanicity and region of residence as it impacts SI specifically among Black older adults. A previous study by Taylor et al. (2021) found African Americans who lived in urban areas had more social interactions with their family members in comparison to those who lived in rural areas. There may greater interactions (and therefore less SI) among Black Americans who live in urban areas because of greater proximity to their family and friend networks than to Black people living in suburban and rural areas.

Region of residence is another factor that may influence SI among Black older adults. Research by Taylor et al. (2016, 2019) found African Americans and African American older adults who reside in the southern United States are less likely to be isolated from family members and friends, from religious congregational members, and are less likely to live alone. Additionally, previous research has noted that there is greater social support and more social interactions among family members living in the South (Sechrist et al., 2007; Taylor et al., 2013, 2021).

### Limitations of the Previous Research and Purpose of the Current Study

Previous research on SI among Black older adults has been underdeveloped with only a few studies. This is important because significant associations between sociodemographic factors and SI are confirmed among older adults generally but remain unconfirmed for older Black adults. Additionally, there are few empirical studies which examine how environmental factors (e.g., neighborhood conditions, urbanicity, and region of residence) influence SI, regardless of race/ethnicity. Lastly, the empirical research to date on SI among Black older adults use older data, and hence, may not accurately reflect current social trends, and/or incorporate new modes of communication.

The purpose of this study is to document the role of sociodemographic and neighborhood/environmental conditions on SI among Black older adults using the 2014 and 2016 waves of the Health and Retirement Study (HRS). Our study extends the SI literature by addressing the knowledge gaps mentioned above (the dearth of studies among Black older adults) and using newer data to examine SI among Black older adults. In addition, previous studies on SI among Black older adults (Taylor et al., 2016, 2019) have used the NSAL, a landmark study of African Americans and Black Caribbeans living in the United States (2001–2003). Thus, the present study uses more recent nationally representative data on Black older adults living in the US. Furthermore, we use a comprehensive constellation of SI indicators (or types of SI), including limited contact from adult children, family members, and friends, a lack of participation in social or religious activities, living alone, being unmarried and combine these indicators using a cumulative index. Lastly, we examine a broad range of sociodemographic and neighborhood/environmental predictors to determine their impact on SI among Black older adults.

## Methods

### Sample

This study utilizes data from the Health and Retirement Study (HRS). The HRS is a nationally representative panel study of adults aged 50 and over living in the United States of America. The HRS began data collection in 1992 and interviews are conducted face-to-face once every 2 years. The HRS Core collects data regarding income, wealth, family structures, health, physical limitations, cognition, and housing. Respondents for the HRS are selected through a complex multistage probability sampling design and the HRS sample is replenished with new respondents once every 6 years. The HRS also oversamples for respondents who identify as Black, Hispanic, and residents in Florida.

In 2006, the HRS started collecting data via the Psychosocial Leave-Behind Questionnaire (LBQ). Topics covered in the LBQ include social engagement and participation, social networks, loneliness, self-rated beliefs, and personality. The HRS LBQ uses a rotational study design in which half of the sample is selected to complete the LBQ survey in 2006, and the other half is selected for the LBQ survey in 2008. For the LBQ, respondents are surveyed once every 4 years; therefore, the 2006 half sample was surveyed again in 2010 and 2014, while the 2008 half sample was interviewed in 2012 and 2016. For more information regarding the HRS Core and the HRS LBQ, please refer to Fisher & Ryan, 2017 and Smith et al., 2013. For this study, the inclusion criteria were (1) participation in the 2014 or the 2016 HRS LBQ and (2) self-identified as Black. This generated a total sample size of 2,323 Black respondents.

### Measures

#### Social isolation indicators

There are 8 dependent variables in the current study to assess SI: (1) isolation from adult children, (2) other family members, (3) friends, (4) disengagement from social or group activities, (5) disengagement from religious services, (6) being unmarried, (7) living alone, and (8) a cumulative measure of SI using an index. For each SI indicator (or type of SI), respondents who were socially isolated were coded as 1, while respondents who were not isolated were coded as 0, as detailed below.

The HRS uses a social network inventory to examine the frequency of social contact with adult children, other family members, and friends. Four different types of social contact were measured, including face-to-face contact, telephone contact, written/e-mail contact, and social media contact. To measure SI from adult children, if the respondents had less than once a month face-to-face, telephone, written/e-mail, or social media contact with their adult children, then they were categorized socially isolated from their adult children (1). If respondents had at least once a month contact with adult children by either face-to-face contact, telephone contact, written/e-mail contact, or social media contact, then they were coded as not socially isolated from adult children (0). The same coding scheme was used for SI from other family members and SI from friends. Disengagement from group or social activities was also a dichotomous variable. Respondents who reported not participating in any of the following social activities: (1) volunteering with youth, (2) doing charity work, (3) attending an education or training course, (4) attending a sports/social group or club, or (5) attending non-religious organizations, were coded as disengaged from social or group activities (1). Disengagement from religious service was also a dichotomous variable and respondents who never attended religious service were coded as being disengaged (1). For marital status, those who were widowed, divorced/separated, or were never married, were categorized as unmarried (1). Living alone was operationalized as a dichotomous measure as either lived alone (1) or with other people (0).

#### Social isolation index

Taking each of the isolation measures detailed above, items were combined into a 7-point SI index. Scores ranged from 0 to 7, with lower scores representing lower cumulative SI and higher scores representing higher SI. Due to the overall distribution, with very few people in the sample with scores of 4 or higher, individuals with a score of 4 or higher for SI were combined into a single category. This SI index and similar versions have been used in many other studies of SI among older adults (Berkman & Syme, 1979; Cudjoe et al., 2020, 2022; Pantell et al., 2013; Schoenbach et al., 1986; Shankar et al., 2011; Taylor, 2020) and among older Black populations (Taylor et al., 2019)

#### Sociodemographic factor

Age was operationalized as a continuous measure. Gender was measured as male and female. Total household income was operationalized as a five-item ordinal variable ($0–$24,999.99, $25,000–$49,999.99, $50,000–$74,999.99, $75,000–$99,999.99, more than $100,000). Educational attainment was a categorical measure with the following categories: less than high school, high school diploma, some college, and Bachelor’s degree or above. Employment status was measured as a dichotomous variable (yes vs. no).

#### Neighborhood/environmental conditions

Region of residence was measured as a categorical variable, and respondents indicated whether they were living in the Northeast, Midwest, South, or West. Neighborhood social cohesion and neighborhood physical disorder were both operationalized as scales with four items. Neighborhood social cohesion was measured by whether the respondent: (1) feels like they belong in this area, (2) can trust others in their neighborhood, (3) other people living in the respondent’s neighborhood are friendly, and (4) whether if other people would help them if they were in trouble. Responses were recorded on a 7-level Likert scale with 1 representing the most positive views of the neighborhood (and greater cohesion) and 7 representing the most negative views of the neighborhood (worse cohesion). Items were reverse coded and averaged together, with higher scores representing greater neighborhood social cohesion. Neighborhood physical disorder was measured by the extent to which (1) the neighborhood has problems with vandalism and graffiti, (2) individuals have a fear of walking home after dark, (3) there are problems with cleanliness of the area, and (4) the quantity of vacant/deserted homes or storefronts in the neighborhood. Responses of the neighborhood physical disorder scale were also coded on a 7-level Likert scale with 1 representing the most positive views of the neighborhood (with less disorder), and 7 representing the most negative views of the neighborhood (greater disorder). Items were averaged together with higher scores representing greater perceived neighborhood disorder. Urbanicity of the environment was coded as urban, suburban, or rural.

#### Health and retirement study wave

Models were adjusted for HRS LBQ wave, the 2014 or 2016 HRS LBQ as a control variable. This measure was included because we wanted to account for any potential differences in SI based on the HRS LBQ wave. Furthermore, controlling for the HRS LBQ wave increases the accuracy of the statistical relationships between the sociodemographic and environmental factors and SI variables in our regression models (Coyle & Dugan, 2012).

### Analytic Strategy

All analyses in the current study used the survey weights provided by the HRS to account for the complex multistage sampling design of the survey to make the data nationally representative. Data were managed in SAS v9.4 and analyzed in Stata v16.1. Table 1 presents frequency distributions and descriptive statistics of the sociodemographic and environmental variables. Table 2 presents the frequency distributions and descriptive statistics of the eight SI dependent variables. Table 3 presents multivariable models for the SI variables. For the first seven isolation indicators (Table 3), we estimated multivariable logistic regression models for each of the dichotomous dependent variables. The SI index was a count measure. Due to over-dispersion, we estimated a multivariable negative binomial regression model. All models included each of the sociodemographic and environmental factors as the independent variables.

**Table 1.** Descriptive Statistics for Sociodemographic Factors and Neighborhood/Environmental Conditions (*N*=2,323).

Variables	Percentage (%)	*N*	Mean	Standard deviation
Gender				
Male	42.32	822		
Female	57.68	1501		
Household income				
Less than $25,000	47.41	1111		
$25,000–$50,000	21.34	527		
$50,000–$75,000	13.32	310		
$75,000–$100,000	5.84	138		
More than $100,000	12.09	237		
Education				
Less than high school	22.26	499		
High school diploma	32.70	764		
Some college	28.34	674		
Bachelor’s or higher	16.70	385		
Employment status				
Not working	60.46	1461		
Working	39.54	862		
Region of residence				
Northeast	15.98	341		
Midwest	16.31	376		
South	59.46	1242		
West	8.24	154		
Urbanicity				
Urban	61.47	1508		
Suburban	19.03	445		
Rural	17.11	334		
Wave				
2014	43.05	1179		
2016	56.95	1144		
Age		2323	64.38	12.00
Neighborhood social cohesion		2243	4.67	1.97
Neighborhood physical disorder		2244	3.29	2.04

*Note*. Survey weights were applied to all percentages and the frequencies are unweighted. Percents and *N* are presented for categorical variables, and *N*, means and standard deviations are presented for continuous variables

**Table 2.** Descriptive Statistics for Social Isolation Indicators and Index (*N* = 2.323).

Variable	Percent (%)	*n*	Mean	Standard deviation
Social isolation from adult children				
Isolated from children	20.94	424		
Not isolated from children	79.06	1823		
Social isolation from other family members				
Isolated from other family members	17.86	360		
Not isolated from other family members	82.14	1920		
Social isolation from friends				
Isolated from friends	21.45	460		
Not isolated from friends	78.55	1824		
Disengagement from social activities				
Disengaged from social activities	27.43	600		
Engaged in social activities	72.57	1703		
Disengagement from religious services				
Disengaged from religious services	18.55	350		
Engaged in religious services	81.45	1962		
Being unmarried				
Unmarried	62.67	1438		
Married	37.33	885		
Living alone				
Living alone	32.33	702		
Living with other people	67.67	1621		
Social isolation index (frequencies)				
0	17.70	417		
1	23.14	509		
2	24.73	576		
3	18.67	392		
4 and above	15.76	277		
Social isolation index (continuous)		2171	1.92	1.72

*Note*. Survey weights were applied to all percentages and the frequencies are unweighted. Percents and *N* are presented for categorical variables, and *N*, means and standard deviations are presented for continuous variables

**Table 3.** Multivariable Models of Social Isolation Indicators and Index.

Variables	Child isolation	Family isolation	Friend isolation	Participation disengagement	Religious disengagement	Unmarried	Living alone	Social isolation index^a^
Age	0.98 (0.96, 1.00)*	1.02 (1.00, 1.03)	1.01 (0.99, 1.03)	1.01 (1.00, 1.03)	1.00 (0.99, 1.02)	1.00 (0.99, 1.02)	1.01 (0.99, 1.02)	1.00 (1.00, 1.01)
Gender								
Male	—	—	—	—	—	—	—	—
Female	0.43 (0.32, 0.57)***	0.48 (0.38, 0.61)***	0.58 (0.44, 0.76)***	0.98 (0.69, 1.39)	0.55 (0.38, 0.81)**	1.69 (1.36, 2.09)***	0.95 (0.75, 1.21)	0.89 (0.82, 0.97)**
Household income								
Less than $25,000	—	—	—	—	—	—	—	—
$25,000–$50,000	0.65 (0.45, 0.95)*	0.62 (0.36, 1.10)	0.64 (0.41, 1.00)	0.70 (0.46, 1.05)	0.62 (0.40, 0.97)*	0.20 (0.14, 0.30)***	0.41 (0.28, 0.60)***	0.68 (0.60, 0.76)***
$50,000–$75,000	0.78 (0.51, 1.19)	0.55 (0.33, 0.93)*	0.66 (0.38, 1.16)	0.50 (0.32, 0.78)**	0.62 (0.34, 1.13)	0.11 (0.07, 0.17)***	0.22 (0.13, 0.38)***	0.59 (0.51, 0.67)***
$75,000–$100,000	0.58 (0.25, 1.34)	0.71 (0.35, 1.47)	0.49 (0.27, 0.88)*	0.75 (0.35, 1.63)	0.55 (0.22, 1.38)	0.04 (0.02, 0.09)***	0.13 (0.07, 0.25)***	0.47 (0.36, 0.60)***
More than $100,000	0.49 (0.25, 0.98)*	0.59 (0.32, 1.06)	0.76 (0.44, 1.31)	0.56 (0.26, 1.21)	0.79 (0.44, 1.39)	0.03 (0.01, 0.06)***	0.09 (0.04, 0.18)***	0.41 (0.33, 0.49)***
Education								
Less than high school	—	—	—	—	—	—	—	—
High school diploma	1.21 (0.82, 1.76)	1.17 (0.81, 1.68)	0.98 (0.67, 1.43)	0.61 (0.46, 0.81)**	0.73 (0.46, 1.15)	0.79 (0.54, 1.17)	1.20 (0.78, 1.84)	0.94 (0.87, 1.02)
Some college	1.00 (0.71, 1.40)	1.68 (1.12, 2.52)*	1.05 (0.72, 1.54)	0.38 (0.24, 0.59)***	0.79 (0.48, 1.28)	1.09 (0.71, 1.67)	1.37 (0.93, 2.01)	0.96 (0.87, 1.05)
Bachelor’s or higher	2.01 (1.22, 3.31)**	2.08 (1.37, 3.17)**	0.66 (0.44, 1.00)	0.19 (0.11, 0.33)***	0.63 (0.35, 1.14)	1.22 (0.73, 2.06)	1.91 (1.29, 2.84)**	1.03 (0.90, 1.17)
Employment status								
Not working	—	—	—	—	—	—	—	—
Working	0.64 (0.42, 0.99)*	0.70 (0.51, 0.95)*	0.99 (0.69, 1.44)	0.56 (0.39, 0.80)**	0.97 (0.67, 1.41)	1.71 (1.28, 2.30)***	1.20 (0.89, 1.63)	0.95 (0.88, 1.03)
Region of residence								
South	—	—	—	—	—	—	—	—
Northeast	1.29 (0.84, 2.00)	1.14 (0.77, 1.71)	0.57 (0.37, 0.89)*	1.14 (0.71, 1.87)	1.94 (01.14, 3.32)*	1.39 (0.93, 2.08)	1.31 (0.86, 2.01)	1.10 (0.99, 1.21)
Midwest	1.26 (0.83, 1.92)	1.34 (0.89, 2.04)	0.80 (0.54, 1.18)	0.94 (0.63, 1.41)	1.08 (0.72, 1.63)	1.24 (0.88, 1.74)	1.71 (1.12, 2.61)*	1.09 (0.98, 1.22)
West	1.83 (0.98, 3.41)	1.50 (0.93, 2.43)	0.76 (0.50, 1.15)	0.64 (0.31, 1.31)	1.76 (0.98, 3.19)	1.18 (0.61, 2.27)	1.32 (0.77, 2.28)	1.17 (1.01, 1.35)*
Neighborhood social cohesion	0.89 (0.76, 1.06)	0.97 (0.83, 1.13)	0.82 (0.72, 0.93)**	0.97 (0.83, 1.13)	1.02 (0.87, 1.19)	1.15 (0.97, 1.37)	1.07 (0.94, 1.22)	0.97 (0.94, 1.01)
Neighborhood physical disorder	0.93 (0.79, 1.08)	1.00 (0.86, 1.17)	0.82 (0.73, 0.93)**	0.95 (0.82, 1.10)	1.00 (0.85, 1.18)	1.07 (0.93, 1.24)	1.04 (0.91, 1.19)	0.97 (0.94, 1.00)
Urbanicity								
Urban	—	—	—	—	—	—	—	—
Suburban	0.74 (0.47, 1.16)	1.04 (0.67, 1.63)	0.94 (0.66, 1.34)	1.04 (0.69, 1.55)	0.91 (0.60, 1.39)	0.63 (0.46, 0.88)**	1.04 (0.76, 1.42)	0.96 (0.88, 1.05)
Rural	1.08 (0.73, 1.63)	1.29 (0.78, 2.12)	0.70 (0.42, 1.17)	0.94 (0.60, 1.49)	1.34 (0.78, 2.30)	0.64 (0.33, 1.23)	0.96 (0.55, 1.70)	0.91 (0.79, 1.06)
Wave								
2014	—	—	—	—	—	—	—	—
2016	1.25 (0.94, 1.67)	1.21 (0.87, 1.68)	0.93 (0.70, 1.23)	1.48 (1.15, 1.92)**	1.55 (1.12, 2.15)**	1.38 (1.01, 1.89)*	1.16 (0.89, 1.51)	1.12 (1.04, 1.22)*

*Note*. All multivariable models account for the complex survey weights of the Health and Retirement Study. With the exception of the social isolation index, all other models were multivariable logistic regression models and present odds ratios with the 95% confidence interval in parentheses. *indicates statistical significance at the .05 level, ** indicates statistical significance at the .01 level, and *** indicates statistical significance at the .001 level

^a^indicates a negative binomial regression was used in the social isolation index model. The last model with the social isolation index present incident rate ratios and the 95% confidence interval in parentheses. All other models present odds ratios and the 95% confidence interval in parentheses.

Multiple imputation with chained equations (MICE) was utilized given the amount of missing data in the sample. MICE allows for the imputation of different types of variables, including nominal, ordinal, continuous, and count variables (Berglund & Heeringa, 2014). In addition to including all of the variables from the regression models in the multiple imputation model, we also included the survey weights and auxiliary variables to the imputation model as recommended by Berglund and Heeringa (2014). We created an additional 20 imputed datasets in total. Each dataset estimates a separate regression model which yields unique parameter estimates and standard errors. The parameter estimates and standard errors from each regression model are then combined to determine statistical significance.

## Results

### Descriptive Statistics

The descriptive results are presented in Table 1. In total, there were 2,323 Black older adults in the 2014 and 2016 LBQ. The average age of the sample was about 64 years old. About 58% of the sample were women. A little less than half the sample (47%) reported income of less than $25,000 per year. Most of the sample either had less than a high school degree (22%) or graduated from high school (33%), were not employed (60%), lived in the South (59%) and lived in urban areas (61%). The mean neighborhood social cohesion score was 4.67 (SD = 1.97) and the mean neighborhood physical disorder score was 3.29 (SD = 2.04). Regarding SI, approximately 21% of respondents were isolated from their adult children, 18% from other family members, 21% from their friends, as well as 27% were not engaged in any group activities and 19% did not participate in religious institutions. The majority were unmarried (63%), and 32% lived alone. The average SI score was 1.92 (SD = 1.72).

### Multivariable Statistics

Multivariable regression results are presented in Table 3.

#### Social isolation from adult children

Women, those who either reported a yearly income between $25,000–$50,000 and $100,000 or more were significantly less likely to be isolated from their adult children than men and those who made less than $25,000 annually, respectively. Respondents with a Bachelor’s degree or more were significantly more likely to be isolated from their adult children compared to those who did not have a high school diploma or GED.

#### Social isolation from other family members

Women, those who reported incomes between $50,000 and $75,000 per year, and those who were employed were significantly less likely to be socially isolated from other family members than men, those with an income less than $25,000 per year, and unemployed/not working respondents, respectively. Those with some college or a Bachelor’s degree or more were more likely to be isolated from other family members than those who do not have a high school diploma or GED.

#### Social isolation from friends

Women, respondents who reported annual incomes of $75,000–$100,000, and those who resided in the northeastern region of the US were significantly less likely to be isolated from their friends in comparison to men, those with annual incomes of less than $25,000, and those who resided in the southern region in the US, respectively. Additionally, those who reported greater neighborhood social cohesion and greater neighborhood physical disorder were less likely to be socially isolated from friends.

#### Disengagement in social activities

Respondents whose yearly income was between $50,000 and 75,000 were less likely to be socially isolated compared to respondents who made less than $25,000 per year. Respondents with a high school diploma or GED, some college, or a Bachelor’s degree or higher, were all significantly less likely to be disengaged from social activities than those who did not graduate from high school. Additionally, those who were employed were less likely to be disengaged from social groups than those who were unemployed.

#### Disengagement from religious service

Women were less likely to be disengaged from religious service than men. Furthermore, those residing in the Northeast region of the US were more likely to be disengaged in religious services than those residing in the South.

#### Being unmarried

Women and those who were employed were more likely to be unmarried in comparison to men and unemployed respondents. Respondents with higher income were less likely to be unmarried compared to those with annual incomes of incomes less than $25,000. Those who reported living in suburban areas were less likely to be unmarried than those who resided in urban areas.

#### Living alone

Respondents who were college graduates (a Bachelor’s degree or more) or those who reported residing in the Midwest were more likely to live alone than respondents who did not graduate from high school or were living in the South. Compared to respondents with annual incomes of less than $25,000, those with greater incomes were less likely to live alone.

#### Social isolation index

Women, those who reported higher income ($25,000 or greater), and those who resided in western states had significantly lower levels of SI compared to men, those with annual incomes of less than $25,000, and those who resided in southern states, respectively.

## Discussion

The purpose of our study was to examine multiple social determinant and neighborhood/environmental conditions related to SI among a nationally representative sample of Black older adults. To the investigators’ knowledge, this is only the second study to examine sociodemographic factors influencing SI among Black older adults using nationally representative data (Taylor et al., 2019), and the first to determine how multiple neighborhood/environmental conditions influence SI in this population. Furthermore, even though our study focuses solely on Black older adults, there are very few empirical studies which have examined the influence of neighborhood/environmental conditions on SI among older adults, regardless of their race and ethnicity. We found numerous factors related to SI among Black older adults, which also varied by the type of SI. These findings also help illustrate that Black older adults are not a monolithic group, and that different indicators of SI among Black older adults are patterned by a variety of factors. We also found there were consistently more sociodemographic factors that were associated with SI in comparison to neighborhood/environmental conditions.

Even though this study examined SI among Black older adults, it is important to note these findings are not unique to this population. Previous investigations of SI among the general population of older adults (Chatters et al., 2018; Cudjoe et al., 2020, 2022) and other communities of color (Jang et al., 2016, 2021; Krause & Goldenhar, 1992; Tibiriçá et al., 2022) have confirmed similar findings. This underscores the fact that certain factors are associated with SI regardless of race, ethnicity, and cultural differences. Our study also confirmed key sociodemographic factors (gender, income, education) that are particularly salient for multiple types of SI among Black older adults that were previously hypothesized to influence SI in this population. These confirmatory findings extend the literature on SI and is a major strength of our study.

### Gender and Social Isolation

Gender appeared to be the most significant factor for SI among Black older adults. More specifically, older Black women were less likely to be isolated than men across multiple indicators of social isolation (e.g., adult children, family members, friends, religious participation, cumulative index of SI); however, older Black women were significantly more likely to be single/unmarried compared to older Black men.

There are a variety of explanations for why older Black men are often more socially isolated in comparison to older Black women. Taylor and Taylor (2018) note that gender differences in SI may reflect differences in socializing behaviors, including women may have a greater tendency to maintain their social connections with members of their social networks. For example, Black women often assume the role of kin keepers in their family (i.e., people who maintain and strengthen family ties; Stack, 1975). Additionally, women tend to engage in more frequent supportive exchanges with members of their social network in comparison to men and derive more social support from their social networks. In contrast, previous work has found men mostly rely on their spouses for social support (Antonucci & Akiyama, 1987; Kahn, 1994; Okun & Keith, 1998; Taylor & Taylor, 2018). Previous research has also found that men experience greater SI than women including among Black older adults and Black populations generally (Taylor et al., 2016, 2019), Asian (Jang et al., 2016, 2021) and Hispanic (Krause & Goldenhar, 1992; Tibiriçá et al., 2022) older adults in the US, as well as among older adults generally (Chatters et al., 2018; Cudjoe et al., 2020, 2022).

### Household Income and Social Isolation

Greater household income was associated with lower likelihood of SI from adult children, from other family members, from friends, and lower likelihood of disengagement from social or group activities. Furthermore, greater income was strongly associated with lower likelihood of being unmarried, living alone, and with lower levels of overall SI. These findings regarding the relationship between household income and SI are also found in previous studies among Black populations and Black older adults (Taylor et al., 2016, 2019), in the general population of US older adults (Chatters et al., 2018; Cudjoe et al., 2020, 2022), among older Asian populations in the US (Jang et al., 2016, 2021), and among older Hispanic populations (Krause & Goldenhar, 1992; Tibiriçá et al., 2022). These findings are noteworthy as they illustrate income is a key driver of SI, regardless of race and ethnicity.

It is difficult to determine the causal pathways between these types of SI and total household income. Reverse causality is very possible here; having greater SI overall, being unmarried, or living alone may be risk factors for lower total household income. Older Black adults who live alone may have lower total household incomes if they are the sole provider and may not also be receiving financial assistance from family members, friends, or other sources. Black older adults who are unmarried may have lower total household incomes due to a myriad of factors including that they may be widowed, are no longer receiving any source of income or survivor benefits from their deceased partner, or may be divorced or separated and thus, would not have access to their former spouses’ incomes, assets, or benefits. Lastly, Black older adults may never have been previously married and therefore would not have a spouse or partner (along with that, their share of the household income) to begin with.

### Educational Attainment and Social Isolation

We found educational attainment had mixed findings with different indicators of SI. Black older adults with greater educational attainment were more likely to be socially isolated from their adult children and from other family members; however, they were more likely to be engaged in social groups and activities. Some previous studies among both the general population of older adults and Hispanic older adults have found that higher education to be associated with lower overall SI (Cudjoe et al., 2020; Krause & Goldenhar, 1992; Pohl et al., 2017; Tibiriçá et al., 2022). Other studies among older adults (Chatters et al., 2018) and Asian older adults in the US (Jang et al., 2016, 2021) found no significant association between education and SI. Conversely, Taylor et al. (2019) also found that greater educational attainment was associated with more SI from neighborhood groups for both African American and Black Caribbean older adults.

We orient the current study findings in that more education may be attributable to greater social mobility among Black Americans. More recently, with desegregation of schools (within both the primary and higher education contexts), Black Americans have been able to access and achieve higher levels of formal education (Administration on Aging, n. d.). With this greater social mobility, the extended family networks of Black older adults may be more dispersed across the United States since they may have greater social and economic capital to move, possibly away from family members. Furthermore, studies on middle-aged to older Canadians and older Swedish adults also found that higher levels of education were associated with greater social isolation (Lundholm, 2015; Menec et al., 2019). As well, Black older adults with higher education were more involved in social groups and activities. Black Americans, and particularly those with some college education or higher, have a rich history of participating in groups, clubs, and organizations (Brown et al., 2012).

### Neighborhood/Environmental Conditions and Social Isolation

Interestingly, environmental factors had limited influence on SI among Black older adults in comparison to the previously mentioned sociodemographic factors. To begin, there were some regional differences in the indicators of SI: in comparison to those who lived in the South, Black older adults living in the Northeast were less likely to be isolated from their friends but were more likely to be disengaged from attending religious services. Black older adults living in the Midwest were also more likely to live alone compared to Black older adults living in the South. Lastly, Black older adults living in the West had greater cumulative isolation than those living in the South. These findings help illustrate how southern Black culture, which places a strong emphasis on family network relationships and participating in religious institutions (Sechrist et al., 2007; Taylor et al., 2021), subsequently can influence SI.

Furthermore, neighborhood social cohesion and neighborhood physical disorder were only significantly associated with SI from friends. Greater neighborhood social cohesion was associated with lower likelihood of being socially isolated from friends, and greater neighborhood physical disorder was also associated with lower isolation from friends. One potential reason for the connection between neighborhood social cohesion and SI is that having greater neighborhood cohesion could be indicative of more positive relationships and greater trust in the neighborhood. This could be associated with less SI from friends, especially if Black older adults view their neighbors as friends. Another potential reason for these findings is that Black older adults may be traveling to visit their friends in different neighborhoods. Black older adults may be more likely to see their friends in neighborhoods with greater social cohesion as opposed to less cohesion.

We also found that greater neighborhood physical disorder was associated with less SI from friends among Black older adults. The research team expected the opposite, in which greater neighborhood disorder would be associated with greater SI because high amounts of graffiti or trash in a neighborhood may serve as a deterrent from wanting to go and interact with other people. This measure of neighborhood physical disorder may be indicative of overall neighborhood population density, in which neighborhoods with greater physical disorder may be associated with greater overall population density. Additionally, older Black respondents in neighborhoods with high physical disorder may also have tight-knit communities and strong bonds between neighborhood members and friends, and they may be working together in efforts to prevent further neighborhood deterioration (Schieman, 2005). Further examination is needed for understanding this relationship.

### Limitations and Strengths

There are several limitations to be noted for our study. We utilized cross-sectional data, hence, it is not possible to establish the time-ordering necessary for causality between sociodemographic factors, neighborhood/environmental conditions, and SI. As mentioned in the discussion section, lower household income could be a result of being unmarried or living alone instead of the other way around. Additionally, it is difficult to determine a prevalence rate of SI due to the dynamic nature of isolation. For example, even if an individual meets the criteria of being isolated across four domains (e.g., the respondent may be unmarried, lives alone, has limited contact from family members, and limited contact from adult children), they could still attend religious services multiple times per week and also meet with their friends multiple times per week as a way to compensate from their lack of social interactions in other domains. This is also why the investigators decided to examine each indicator of SI separately to determine what sociodemographic and environmental conditions influence individual types of SI. Additionally, this points to future research and conducting a latent profile analysis to determine if there are certain profiles of SI among Black older adults, and which of these profiles are most detrimental to health (Lincoln & Nguyen, 2021; Nguyen, 2017, 2021).

We used neighborhood social cohesion and neighborhood physical disorder which are perceived/subjective measures of the respondents’ neighborhoods. This means that two different respondents can view their neighborhoods in very different ways, and this can be influenced by how respondents feel about their neighborhood. Nevertheless, previous studies have found perceived neighborhood conditions have a significant association with health (Wen et al., 2006). This is an important step in determining how neighborhood conditions shape SI, and future studies can also consider using both objective and subjective measures of neighborhood social cohesion and physical disorder. Fourth, the HRS does not delineate different categories of Black people, whether they be African American or Black Caribbean. Previous studies have found that there are important ethnic differences in SI among Black older adults and among Black men (Taylor & Taylor, 2020; Taylor et al., 2019).

Despite these limitations, there are many notable strengths to the study. Our study examined multiple forms of SI among Black older adults, demonstrating the forms and expression of SI are not monolithic in this population. Findings from our study are also important regarding the development of SI interventions for Black older adults. For example, if an individual is experiencing SI from their family members, there could be different intervention strategies utilized compared to an individual who does not participate in group or social activities. Given this is one of the first studies to use nationally representative data to examine both sociodemographic and neighborhood/environmental conditions, additional research is warranted to determine the causal pathways of SI. How do specific neighborhood/environmental conditions, such as the presence of a senior center, park, or neighborhood church, buffer or increase SI? Do sociodemographic factors mediate or moderate the relationship between neighborhood/environmental conditions and the multiple forms of SI? If neighborhood/environmental conditions change over time, does this also affect SI among Black older adults? We hope our study will spur further research questions and will be foundational for building the empirical literature for SI among minority populations.

## Conclusion

The population of Black older adults is increasing rapidly, and it is important to consider that many from this population may be aging alone, without the support of family members, friends, or participating in groups or social activities. We are hopeful the findings from this study will serve as an impetus for further research in this area, for developing interventions and tools to help socially isolated Black older adults to become re-integrated within their social networks (or with the formation of new social networks), or for developing policies and programs to reduce SI in this population.
